# Supplementary material for: Causal Relationship between Aging and Anorexia Nervosa: A White-Matter-Microstructure-Mediated Mendelian Randomization Analysis
Source: Biomedicines. 2024 Aug 16;12(8):1874. doi: 10.3390/biomedicines12081874 (PMC11351342; doi:10.3390/biomedicines12081874)
Supplement: Supplementary file 1 [file biomedicines-12-01874-s001.zip › Supplementary Figure/Figure S8/Leaveoneout plot of MR analysis for TL to ubm-b-2034.pdf]

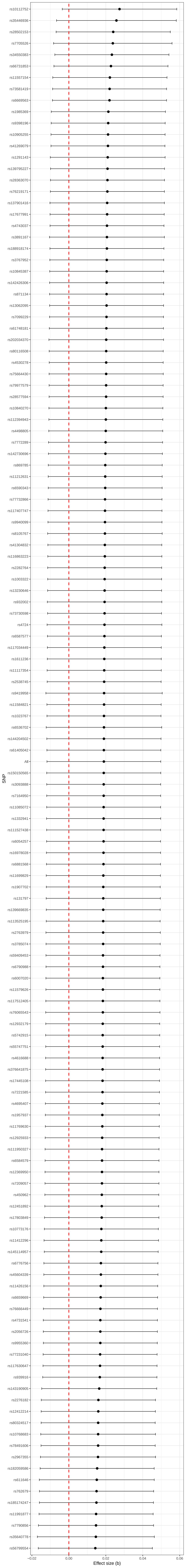
rs10112752
rs35446936
rs28502153
rs7705526
rs34550383
rs66731853
rs11557154
rs73581419
rs6669563
rs1985369
rs9398196
rs10905255
rs41269079
rs1291143
rs139795227
rs28363070
rs76219171
rs137901416
rs17677991
rs4743037
rs3891167
rs188918174
rs3767952
rs10845387
rs142426306
rs871134
rs13062095
rs7099229
rs61748181
rs202034370
rs80116508
rs4530278
rs75664430
rs79977579
rs28577594
rs10840270
rs112394943
rs4498805
rs7772289
rs142730696
rs869785
rs11212631
rs6590343
rs77732866
rs117407747
rs9940099
rs8105767
rs41304832
rs116863223
rs2282764
rs1003322
rs13230646
rs932002
rs73730598
rs4724
rs6587577
rs117034449
rs1611236
rs11117354
rs2538745
rs9419958
rs11584821
rs1023767
rs6536702
rs144204502
rs61405042
All
rs150150565
rs3093888
rs7164950
rs11085072
rs1332941
rs111527438
rs6054257
rs16978028
rs6881568
rs11699829
rs1907702
rs131797
rs139669835
rs113525195
rs2763979
rs3785074
rs59409453
rs6790988
rs6007020
rs11579626
rs117512405
rs76065543
rs12932179
rs5742915
rs55747751
rs4616688
rs376641875
rs17445108
rs7221585
rs4695407
rs1957937
rs11769630
rs12925933
rs111950327
rs6584579
rs12369950
rs7209057
rs450962
rs12451892
rs17803849
rs10773176
rs11412296
rs145114957
rs6776756
rs45604339
rs11426156
rs6659669
rs76666449
rs4731541
rs2056726
rs9955360
rs77231040
rs117630647
rs939916
rs143190905
rs2276182
rs12412214
rs80324517
rs10768683
rs78491606
rs2967355
rs182059586
rs611646
rs762679
rs185174247
rs11991877
rs7790856
rs35640778
rs56799554
SNP
−0.02
0.00
0.02
0.04
0.06
Effect size (b)
